# Walking through life with cerebral palsy: reflections on daily walking by adults with cerebral palsy

**DOI:** 10.1080/17482631.2020.1746577

**Published:** 2020-04-02

**Authors:** Beate Eltarvåg Gjesdal, Reidun Jahnsen, Prue Morgan, Arve Opheim, Silje Mæland

**Affiliations:** a Department of Health and Functioning, Western Norway University of Applied Sciences, Bergen, Norway; b Department of Global Public Health and Primary Care, University of Bergen, Bergen, Norway; c Department of Clinical Neurosciences for Children, Oslo University Hospital, Oslo, Norway; d Institute of Health and Society, CHARM, University of Oslo, Oslo, Norway; e Department of Physiotherapy, Monash University, Victoria, Australia; f Institute of Neuroscience and Physiology, Rehabilitation Medicine, the Sahlgrenska Academy, University of Gothenburg, Gothenburg, Sweden; g Region Västra Götaland, Habilitation & Health, Gothenburg, Sweden; h Research Department, Sunnaas Rehabilitation Hospital, Nesoddtangen, Norway

**Keywords:** Adults, cerebral palsy, walking, gait, reflections, interview

## Abstract

**Purpose**: Walking is a major target in childhood physiotherapy for children with cerebral palsy (CP). Little information exists on the importance or value of walking when these children grow up. The aim of this study was to explore personal reflections on daily walking by adults with CP.

**Method:** Semi-structured individual interviews were conducted and analysed with systematic text condensation, a four-step thematic cross-case analysis.

**Results**: Eight ambulatory adults (26–60 years, four women and four men) with CP were interviewed. Almost all had experienced deteriorated walking ability in adulthood and reported that walking was restricted and affected by intrinsic features, such as pain, fatigue, reduced balance and fear of falling. Extrinsic features such as being looked at due to walking abnormality and environmental factors, such as seasonal changes affected their free walking and was common. Some had accepted using mobility aids for energy conservation.

**Conclusions**: Both intrinsic and extrinsic factors influence walking in adults with CP. Reflections by the adults with CP suggest these features may reduce participation in public spaces and potentially increase acceptance and use of mobility aids.

## Introduction

Walking is an important activity for health and well-being (Nordh et al., 2017; Stamatakis et al., 2018) and slow walking speed greatly increases the risk for social participation limitations in older adults (Warren et al., 2016). Several populations with neurological conditions are known to demonstrate impaired walking due to factors, such as reduced muscle strength, joint range of motion, coordination and motor control, leading to increased spatiotemporal asymmetry or unsteadiness (Mahlknecht et al., 2013). Such impairments, may in turn, restrict mobility by limiting walking distance, walking speed or the ability to manoeuvre in various environments and contexts. Cerebral palsy (CP) is one such condition that is associated with impaired walking. CP is caused by non-progressive brain damage acquired early in life, but living with CP is not viewed as being in a static condition (Mutch et al., 1992; Rosenbaum et al., 2007). Motor function in persons with CP is highly variable. It has been reported that 70% of those diagnosed with CP will learn to walk, although later than typically developing children (Andersen et al., 2008).

Many children with CP undergo extensive rehabilitation during their childhood aimed at acquiring functional abilities (Moll & Cott, 2013). Being able to walk might contribute to independence in daily activities (Andersson & Mattsson, 2001), and has been described as an important rehabilitation domain both by parents of children with CP and health care professionals (Bottos et al., 2001; Vargus-Adams & Martin, 2011). Despite extensive rehabilitation, including training of motor functions during childhood, persons with CP have reported negative musculoskeletal changes and subsequent reduction in functional status (Turk, 2009). This has been described as a “general slowing down” (Moll & Cott, 2013), and adults with CP have reported twice as much pain, and significantly more fatigue than the general population (Jahnsen, Villien, Aamodt et al., 2004; Jahnsen et al., 2003). Recent studies found that adults (18–30 years of age) with CP are approximately seven times more likely to have musculoskeletal morbidities, such as osteopenia, osteoporosis, osteoarthritis and rheumatoid arthritis compared to typically developing young adults (D. Whitney et al., 2018), high risk of musculoskeletal morbidities is also reported across all age groups (D. G. Whitney et al., 2018). These and other impairments suggest the need for continuous healthcare into and throughout adulthood. However, regular follow-up and access to rehabilitation may be experienced as difficult to access or even absent by adults with CP (Morgan et al., 2014).

Studies have shown that 25–58% of adults with CP experience decline in mobility (Bottos et al., 2001; Himuro et al., 2018; Morgan & McGinley, 2013). Deterioration of walking ability has been described as early as mid-`30 s and `40 s (Moll & Cott, 2013; Opheim et al., 2013) in contrast to typically developing persons who report deterioration after age 75 years. In CP, those with worse initial walking ability, being bilaterally affected, and reporting higher levels of fatigue experience greater deterioration (Morgan & McGinley, 2013). Furthermore, deteriorated walking ability has also been found to be associated with more pain and self-reported reduced balance (Opheim et al., 2009). The perception of deteriorated walking is subjective (Opheim et al., 2012, 2013), however a Kaplan-Meier estimate including 105 adults with CP illustrated a marked decline evident around the age of 35 years (Opheim et al., 2009). To date, we have little knowledge about how walking deterioration influences the day-to-day lives of an adult with CP, suggesting a need to explore this in more detail using qualitative methodology (Opheim et al., 2013).

This study aimed to explore personal reflections on daily walking by adults with cerebral palsy.

## Materials and methods

Based on the paucity of first-person reflections on daily walking in adults with CP, we designed an exploratory, inductive qualitative study using semi-structured individual interviews.

### Participants

Ambulant adults with CP (>18 years) living in the community, of any CP subtype, with GMFCS Level I–III (Livingston et al., 2007), who had completed secondary school, able to partake in a conversation and provide informed consent, were invited to participate. Information about the study was sent to the Secretary General of the Norwegian CP-association, who forwarded the email to adult members of the regional CP-association. Information was also posted on the Norwegian CP-association and Western Norway University of Applied Sciences Facebook page with encouragement for others to share using snowball sampling to maximize the spread of information to a population who were challenging to reach (Sedgwick, 2013). Further, written information regarding the study was made available at a Norwegian training centre for adults with CP and the regional hospital. Those interested in participating contacted the first author, and after confirming inclusion criteria, an interview appointment was scheduled.

Eight adults with spastic CP, four men and four women, aged 26–60 years, who had completed secondary school, were included in the study. Six participants were students and/or were employed, and two were receiving disability benefits. All participants resided in South-Western Norway. A written consent form with information about the study was distributed by email. None of the participants had met or had contact with the interviewer in any rehabilitation or treatment setting previously.

### Ethics

Ethical approval was granted by Regional Committees for Medical and Health Research Ethics (REC West Norway) (2018/349).

### Data collection

**Figure 1.**
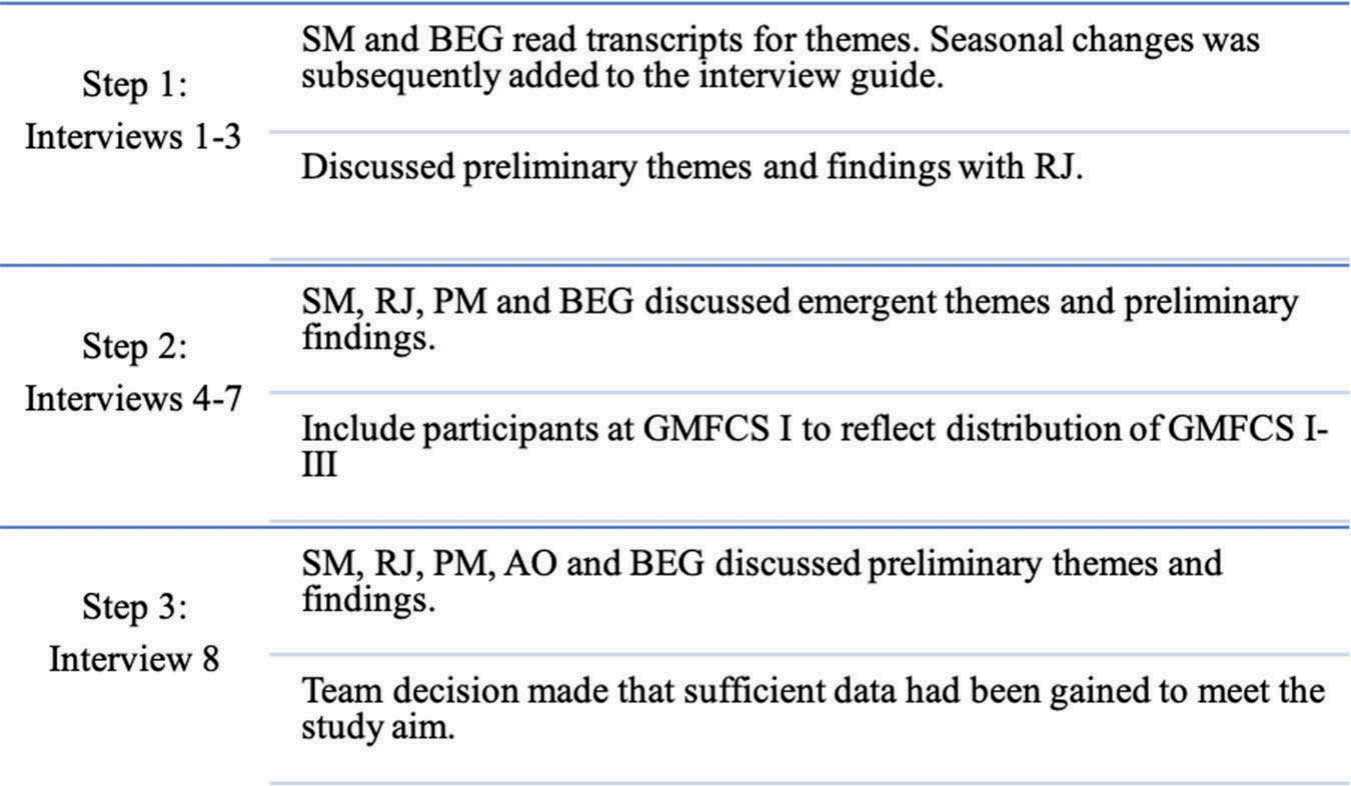
Data analysis flow chart. Step 1: After three interviews SM and BEG read transcripts for themes and discussed them with RJ. Step 2: After seven interviews SM, BEG, RJ and PM found it necessary based on themes and findings to include GMFCS I. Step 3: The team concluded that information power was reached for the material

The individual interviews were semi-structured and took place at a mutually convenient location. The interviews lasted from 28 to 68 minutes (mean 50 minutes). Consent to audiotaping and verbatim transcription was given before the interview commencement. It was emphasized to the participants that no right or wrong answers existed, and the best information they could give was sharing their reflections regarding daily walking. The first author conducted the interviews starting with an overarching question “Can you share your experience on how it is to walk with CP?” The dialogue evolved reflections on the importance of being able to walk on a daily basis, and keywords that formed the interview guide were based on the research question: a normal day, falling, the wish/need to do exercises targeting walking, overuse injuries, energy consumption, changes experienced in adulthood, fatigue, inpatient rehabilitation as adults, and additionally seasonal changes was included following interviews 1–3 (Figure 1). The interviewees were only interrupted when a clarification was needed.

We included only current walkers (GMFCS level I–III) and therefore gained a sample with specific and recent experiences of daily walking to share. We explored reflections regarding walking in persons with CP in adulthood and assessed the quality of the dialogue to be particularly strong in seven out of eight interviews (Malterud et al., 2015). Questions such as “*What are your thoughts about this?”* were asked to encourage further reflections. The data collection process was an iterative process involving all authors at different steps described in Figure 1. Traditionally *saturation* is used to describe when further empirical data does not add more to the data (Malterud, 2012), however in this exploratory study we used *information power* to guide our data collection. Our ambition was not to cover the whole range of the phenomena (walking), but rather to present new insights into daily walking and identify emerging information relevant to the study aim) (Malterud et al., 2015). In the iterative process we discussed the study aim, sample specificity, use of established theory, quality of dialogue and analysis strategy that all influenced the strength of the data and hence information power (Malterud et al., 2015). Through this ongoing evaluation we identified that we had sufficient data to answer the research question after eight interviews.

### Data analysis

Data were analysed with systematic text condensation (STC), a four-step thematic cross-case analysis that offers a framework befitting the explorative aim of the study (Malterud, 2012). The first (BEG) and last author (SM) conducted the first three steps of STC, reading the data material to gain an overall impression, coding meaning units that represented reflections on walking in adulthood and amalgamating the content from meaning units. In the first steps, we received input in the analysis process from RJ and AO. In the last step, when the material was written in third-person format, we translated the text to English, and all authors worked together in an iterative analytical process. A more detailed description of STC can be found elsewhere (Malterud, 2012).

## Results

Both intrinsic and extrinsic factors influenced reflections on daily walking. Three types of intrinsic factors were described: reduced functional capacity, daily walking as energy-demanding, but chosen activity and reduced balance hinder free walking. The extrinsic factors influencing daily walking were: attracting attention from onlookers due to walking variation, seasonal changes challenging walking and mobility, and accepting walking aids.

### Intrinsic factors and their influence on walking

#### Reduced functional capacity influenced walking

Many participants had experienced adverse physical changes in adulthood and pondered whether these changes came about because they had used their body in an “unsound manner” for years, without preventive measures. Several participants described pain as a factor that influenced their current walking ability negatively. One person described how walking resulted in increasing back pain and subsequent reduced the capacity to walk. A participant in the 30 s, regularly working long hours, was reluctant to realize that reduction in walking ability could happen, although acknowledging a strong message from a physiotherapist to “stop working so much in order preserve current walking ability”. Some participants reported that bodily changes and subsequent mobility restrictions were insidious and slowly emerging in onset. In contrast, others described that changes in mobility came suddenly like “an avalanche”, exemplified by a participant who recalled the time when the ability to step up onto a curb without support was lost. Alterations, such as reduced muscle strength and joint flexibility may have led to mobility restrictions, which in next turn may lead to further functional decline in a vicious circle. Also, when bodily changes forced the participants to inactivity, this could lead to a feeling of “not being good enough”. One participant described how the enforced inactivity had resulted in having to cope with the subsequent feeling of being “lazy”.

Most participants described restrictions in more complex walking tasks, like during uphill or prolonged walking distances, resulting in reduced walking speed. One young participant described that spasms and foot muscle soreness increased when walking long distances and increased limping was associated with tiredness. A dilemma mentioned by several participants was that the “less affected” body side got tired when trying to offload the “more affected” side. Another participant described having to stop going to concerts due to the burden of prolonged standing. Even though trying to adapt to the situation by leaning towards a wall, it became too strenuous, and it felt like the inability to offload body weight interrupted the concert experience. Other participants described difficulties with, and some subsequent cessation of different activities, such as swimming, riding a bike, and domestic activities like vacuuming and picking up objects from the floor. Some participants chose to remain very active by doing activities, such as hiking and walking the dog despite increasing aches and pain. One of the participants shared strategies to continue with daily tasks when the pain set in:

“I sit down as soon as I can and work primarily in sitting. I do this because the back gets me, and my feet are in constant tension (…). I actually feel my back now after this marginal amount of walking. So, I take a lot of paracetamol. Yes, this is a great burden” (Participant 2).

#### Daily walking as an energy-demanding, but chosen activity

Participants described a range of experiences associated with maintaining walking ability. Some participants reported that to minimize the negative impact on social activities, they would proceed with the task as planned, although it was a physically challenging one. One participant justified the strenuous effort of walking alongside the partner on a hike or when shopping, because it was important to their relationship—even though eventually ending up walking behind the partner. For some participants, a good night`s sleep was enough to re-energize for the next day. One young participant compared living with CP to “living like an athlete”, always pushing their body to the limit. Still, many participants felt being stubborn and having a “strong will” as beneficial and essential in order to maintain their walking ability for as long as possible. One participant described the importance of walking as:

“My whole life is based on my ability to walk, then I am free to do what I want without significant help from others. It is important to have a minimum of walking function that can take me places that are not accessible (with mobility aids)” (Participant 5).

Walking, training/exercising, studying and working were all mentioned as energy-consuming activities that made them reflect upon the question “what is the smartest thing to do?” Increased energy expenditure during daily activities directed them to choose the least energy-consuming alternative. One participant described choosing the elevator instead of the stairs from the parking lot, in order not to arrive at the office in a sweat. Another participant told about sometimes having to push oneself beyond the limits in order to keep up, for instance, being able to attend afternoon soccer training with the children. Other strategies that were used to cope with the limited energy could be locating a car park within a short walking distance to the target destination, and also always looking for a chair or bench when walking. The participants found that the need for energy conservation took time to accept and planning for having sufficient energy for the whole day was a concern for all of them.

#### Reduced balance restricted free walking

The participants generally experienced that their balance had become reduced and that this was strongly connected to how they walked. All of the participants had fallen previously, and the reduced balance limited them with regards to walking distance and also a greater need for taking breaks and sitting down. Many participants reported that their balance had become worse in adulthood. The problems became evident in daily activities, and they described activities like ascending and descending stairs, jogging, dancing and walking in shops and malls, as problematic. They also found it more embarrassing and “scarier” to fall with increasing age. A younger participant reflected that this might be the case because it felt more “natural” to fall as a child or youth. Although some participants fell infrequently, one person described the feeling of always being nervous when walking downhill. Another was nervous about being unsteady when walking on stones and pebbles. On occasions, failing to consider fatigue, a grass tussock was enough to provoke a fall. The same participant also reported that trying to hide the walking difficulty while walking alongside someone, would almost certainly lead to a fall.

Several participants reported that at home there was always something to grab hold of when getting up after falling, in contrast to outdoors. Falls were, therefore, easier to cope with at home.

Walking up or down stairs could be challenging, and one participant reported fear of being unsteady, and thus consequently did not carry anything when descending stairs. Crowded places, like shopping malls, were avoided by many participants, since they needed more space to move freely. One participant described a sense of being “carefree” when walking in the mountains or in the woods. When being outdoors, some participants reported that they could not get up without help from others after falling and therefore were reluctant to go out. This is how one participant described the change in adult life:

“Before, I used the crutches to get up after falling, but that is too difficult now, and I cannot manage to get back on my feet, and that makes it scarier to fall. Indoors, I can climb up against something, but outside I’m just lying there like a seal in need of help. That was how I was forced to recognize that I cannot walk that much anymore. Because if I walk, then I fall, and this made it easier to accept using the wheelchair. I could not stand having it like that, I could not stand falling all the time” (Participant 1).

All these intrinsic factors precipitated the need for contact with health care professionals, as they were not prepared for these changes even though some had received information about the late effects of CP from older friends with CP and health care professionals. While some participants, in the beginning, struggled to manage these changes, others felt that an awareness of the late effects allowed them to accept the bodily changes and reduced functional capacity more smoothly. In contrast, one participant felt that his/her walking ability had been stable and even improved since childhood and had not noticed any deterioration.

### Extrinsic factors and their influence on walking

#### Attracting attention from onlookers due to walking variation

The independent walkers (n = 6) experienced walking as “natural” despite their CP; however, the visual influence of their abnormal walking pattern on onlookers remained a source of concern. Many participants described distress when onlookers commented on the appearance of their walking, although they reported that these comments affected them more when they were younger. One young participant talked about the self-induced reduction in running speed when passing others because the movement anomaly was more visible when running faster. Another young participant described a mismatch between own walking self-confidence and the visual impact of the walking pattern on others, reporting that onlookers were “terrified” when watching the participant descending stairs, a task that was felt confident by the participant. One participant shared the emotions that people’s looks provoked:

“When hiking or walking downstairs I slow down, and the feeling of people looking at me being different hits me. Meeting new people is always challenging, as I fear them judging me on my walking handicap. I wish they could see «me» and not the other (CP)” (Participant 8).

#### Seasonal changes challenges walking and mobility

Walking on icy surfaces made them use more energy and time to ensure safe footing, and as a result, they had to add extra time to get to appointments in time. One participant who resides near a bus stop, still chose to use the car on icy days despite additional expenses with tolls and parking fees, because of fear of slipping and falling. Another participant reported a preference for in-patient rehabilitation stays during winter because being away from home for a couple of weeks, meant not having to worry about going outside for grocery shopping and other errands. Participants who occasionally used a wheelchair felt frustrated regarding inadequate wheelchair design for winter conditions, leaving them feeling unsafe and restricted in their mobility. Therefore, summertime was preferable for many participants.

“On cold days I can go as far as usual, but I can stumble more because I do not lift my legs properly and then there is a big chance that I fall or slip. There have been some falls on the ice to say the least” (Participant 6).

#### Accepting walking aids

This theme overlaps the prior intrinsic themes of balance, falling and energy-saving strategies, as it describes how accepting walking aids can be helpful when coping with these issues. Walking aids, like a crutch or stick, was perceived as helpful by those reporting to be unsafe when walking. Some participants kept a crutch in the car and thought of it as an assistance in balance function, rather than an aid to increase walking distance. Some felt insecure, even when using a crutch. Big and heavy customized shoes were mentioned as a contributing factor to increased falls risk when walking. This footwear could be challenging during summer as foot sweat and sand between toes could be enough to disturb free walking. One participant described how the partner persuaded to get a disability access-parking permit in order to increase community access and mobility. After the initial reluctance, the participant admitted that this was beneficial for increasing social participation. Several others also had experience with the time-demanding processes of accepting the use of various types of mobility aids and special disability permits. This process of acceptance often started when activities of daily living suddenly became more challenging with increasing age, and the technical aids enabled them to pursue their activities. Some participants used the aids both as an energy saving strategy and to prevent falling. One young participant reported that it was important to keep the walking function “as good as possible for as long as possible”, and therefore used the wheelchair at times in order to save energy during parts of the day. Many of those who started using a walking aid, found it better than first assumed and expected, because suddenly they had more energy to “do other things”. However, the experience of accepting walking aids was described like this by one participant:

“This is how I was forced to figure out I could not walk as much anymore, as I get tired and start falling. I could not bear to have it this way, and this made it easier for me to accept the wheelchair, I did not want to constantly fall” (Participant 1).

Nearly all participants described deterioration in walking ability associated with increasing age, and that it was easier to speak up and inform those around them about their mobility limitations when they became older. Exercises to maintain walking ability was experienced as important for them, although most participants expressed scepticism as to whether they could improve their walking function. Still, they emphasized the importance of maintaining some form of walking function for as long as possible.

## Discussion

This qualitative study provides reflections regarding daily walking and changes in walking ability in adulthoods from a sample of ambulant persons with CP. The main findings were that adverse bodily changes, such as increased pain and enforced energy-saving strategies, were together with reduced balance, reported to affect daily walking performance. These intrinsic factors, along with extrinsic factors, such as seasonal challenges, may restrict daily free walking. Consequently, mobility aids might be more willingly accepted and used in adulthood in order to save energy for social participation. Lastly, walking “differently” may force those with CP to accept being stared at when walking in public spaces.

### Intrinsic factors

Although it is well known that 25–58% of adults with CP experience walking decline (Morgan & McGinley, 2013), this study revealed not all felt prepared for these changes. Some had incidentally “heard” of such decline through friends or health care professionals and interpreted this as a result of having used their body in an “unsound” manner over time. This variation in knowledge and awareness may come as a result of lack of access to health care professionals with knowledge about adult CP disability (Andersson & Mattsson, 2001). Patient education program is used in the computing context may increase knowledge about future disability and increase autonomy in treatment decisions as seen in patients with multiple sclerosis (Köpke et al., 2009) however one may argue that increasing disability and increasing fear of the future may result in self-restriction of activities as described in older adults with CP (Morgan & McGinley, 2018).

Our findings about how pain and fatigue restricted the ability to participate in activities of daily living, has previously been found in cross-sectional studies (Jahnsen, Villien, Aamodt et al., 2004; Jahnsen et al., 2003; Opheim et al., 2009). However, in this study we got in-depth descriptions and reflections on how this impacts their lives and the range of decisions and dilemmas they had to face during activities that able-bodied persons do not have a conscious adherence to. The current study found that management strategies, such as planning ahead and activity modification helped them to cope with pain and fatigue and this is in line with a previous study (Brunton, 2018). This may potentially empower them to keep walking and hence remain socially active and participating.

All participants in this study had experienced falling when walking and felt more embarrassed with falling as they grew older. Falls are common in adults with CP across all GMFCS levels (Morgan & McGinley, 2013). Several participants described being nervous about falling, supporting previous research reporting a greater fear of falling in adults with CP than among older people without disabilities (Morgan & McGinley, 2013). Results from the current study indicate that adults with CP may refrain from going out due to fear of falling. Both falling, and the fear of not being able to get up again, may have negative impact on independence and participation. Negative changes in balance function may seem to have the same negative effect as participants shared stories and reflections about them reducing or stopping activities. These findings shed light and depth to previous findings from quantitative research (Jahnsen, Villien, Egeland et al., 2004; Opheim et al., 2012), supporting the need for life-long follow-up regarding balance and mobility. Our results may assist clinicians become aware of which walking activities adults with CP sacrifice, enabling a person-centred approach to tailoring follow-up interventions.

### Extrinsic factors

Adults with CP reported experiencing unsolicited attention and comments about the appearance of their walking, but such comments had usually affected them more at a younger age. It has previously been reported that youth with CP have experienced stress, depression and anxiety related to bullying from peers at school or social isolation linked to the bodily differences (Lindsay, 2016). As a youth, they “longed” to be “normal” and were frustrated with having to cope with bodily differences resulting in isolation from peers (Lindsay, 2016). The results from this qualitative study support this premise with acknowledgement of “being different” but suggest some decline in the impact of such adverse comments as individuals with CP age.

Physical environmental factors, like cold weather and icy ground, were reported to make it more energy-demanding to walk, also they felt that their muscles got stiffer. Winter conditions are a common environmental factor for Norway and many other northern hemisphere countries, with cold winters influencing walking negatively. The frustration with continually having to plan ahead in order to go to different places, has been expressed in youth with CP (Palisano et al., 2009) and was reinforced during winter conditions by participants in this study. Both independent and community-based strategies to facilitate safe access outside the home during adverse weather conditions should be explored further.

Concurrent with an awareness of adverse bodily changes, some participants reported having to accept mobility aids to assist with balance, prevent falling and save energy. The process of acceptance of these aids often started when daily activities suddenly became more challenging with increasing age, and mobility aids enabled them to pursue their activities—albeit some required significant persuasion from the family. Some participants in this study used a wheelchair as an energy-saving strategy, similarly reported by participants in a study of Stewart et al. (2012) who explained how such choices often were guided by weighing the trade-offs inherent in their social participation. However, these participants were representative of all GMFCS levels. The use of assistive devices has also been linked to stigma threatening adults with CP’s ability to maintain a feeling of being “normal” (Lindsay, 2016). This premise was echoed by some participants in this study.

## Strengths and limitations

A qualitative design was considered the most appropriate approach to explore reflections of daily walking in adults with CP, as this research method is suitable when exploring human experiences (Malterud, 2001). The interviewer and respondents had never met before, and this might have strengthened the confirmability in how the respondent was not eager to please the interviewer in any health-related issues (Richards & Emslie, 2000). BEG and SM wrote down their expectations about potential findings before data collection and BEG kept a reflexive log that she discussed with SM in the process. Two authors (BEG and SM) took part in all steps of the analysis and focused on distinguishing between what we thought we would find and what was found, to commit to reflexivity (Malterud, 2001).The focus on walking in everyday life and the wide age distribution of our purposive sample is considered a strength as previous research has shown that changes may occur both in early and late adulthood. This range is used in previous research on adults with CP (Bottos et al., 2001; Himuro et al., 2018; Morgan & McGinley, 2013) thus strengthening the transferability of the results. Finally, commonality of experience in walking decline and managing walking whilst ageing was reflected across gross motor function levels (GMFCS Levels I–III), rather than reflective of a single GMFCS Level (e.g., Level III). This may be an area for greater exploration. As this study is based on the reports of eight adults with CP, it does not permit any conclusions or generalization however about the impact of age, gender, living situation or GMFCS level on walking ability. A final point is that results may need to be considered in the context of Norwegian healthcare, geography and climate.

## Conclusion

Reflections from adults with CP shed light on how both intrinsic and extrinsic factors affected their daily walking. Whereas intrinsic factors, such as pain, reduced capacity and fear of falling resulted in reduced participation in public spaces and encouraged the use of walking aids. Extrinsic factors such as being a target for curious onlookers’ and challenges of a harsh winter season challenges balance and free daily walking more than usual. These factors aid our understanding of how walking ability influences daily life of adults with CP and emphasize the need for lifelong follow-up by healthcare.
